# Collaborating With Young People: Identifying the Barriers and Facilitators in Co‐Designed Research

**DOI:** 10.1111/hex.70308

**Published:** 2025-05-27

**Authors:** Briony Lipton, Helen Dickinson, Jodie Bailie, Belinda Hewitt, Anne Kavanagh, Zoe Aitken, Marissa Shields

**Affiliations:** ^1^ School of Business UNSW Canberra Canberra Australian Capital Territory Australia; ^2^ University Centre for Rural Health, The University of Sydney Sydney New South Wales Australia; ^3^ School of Social and Political Sciences The University of Melbourne Melbourne Victoria Australia; ^4^ Melbourne School of Population and Global Health The University of Melbourne Melbourne Victoria Australia

**Keywords:** codesign, coproduction, participatory research, young people

## Abstract

**Background:**

Undertaking collaborative research with young people could result in more relevant research and policy. However, there remains a limited understanding of the barriers and facilitators to meaningfully working with young people. This scoping review aimed to identify the barriers and facilitators of engaging young people in codesign research processes.

**Methods:**

This scoping review drew on methodological guidance from JBI. Searches were conducted in Proquest, Scopus, Informit, and Science Direct for relevant peer‐reviewed publications for the period of January 2003–August 2023. Publications were included if they used the term codesign and/or related participatory research methods with young people aged 15–24 years. Two independent reviewers undertook all stages of screening and data extraction, with consensus reached at each stage of the study. Qualitative content analysis was used to group results into key themes.

**Results:**

The search yielded 1334 publications, with 41 meeting inclusion criteria. Publications varied with respect to the age range of included young people, and focused on a variety of populations, including young people with mental ill‐health, with disabilities, First Nations youth, and young people involved with specific services or programs. In analyzing the barriers and facilitators to engaging young people in co‐designed research we found overall that facilitators included consistent funding, dedicated staff, flexible methods, and youth involvement as co‐creators, supported by community networks and extended timelines. Key barriers were limited resources, staff capacity, and logistical challenges like recruitment, transportation, and external responsibilities, which hinder participation.

**Conclusions:**

In conclusion, there is no universal approach to codesign; instead, every project depends on the interplay of various factors. Elements such as resources, communication, process, agency, investment, and relationships can either facilitate or hinder progress, depending on how they are handled. A project that effectively incorporates these interconnected and interdependent factors is much more likely to foster meaningful and lasting collaboration.

**Patient or Public Contribution:**

This study was a scoping review and did not involve patients, service users, caregivers, individuals with lived experience, young people, or members of the public in its design, conduct, analysis, interpretation, or preparation. While the nature of the research—focused on synthesising the existing literature—did not necessitate direct involvement, the absence of young people's participation is acknowledged as a limitation. Nevertheless, the findings are intended to inform future participatory research practices that centre and engage young people and other stakeholders in meaningful, collaborative ways.

## 1 Introduction

Codesign is a collaborative research method that actively involves end users—such as community members or service recipients—as partners in the development of interventions, programs, or policies [1, 2, 3]. Grounded in the belief that those directly affected by an issue possess unique insights, codesign aims to create more relevant, effective, and sustainable solutions [4]. This is especially true when engaging young people, who bring creativity, fresh perspectives, and firsthand knowledge of the challenges they face [5, 6, 7].

However, codesign with young people poses distinct challenges. Power imbalances, communication barriers, and differing expectations between researchers and youth can hinder collaboration [8, 9, 10]. Logistical issues—such as time constraints, accessibility, and the need for skilled facilitation—can also obstruct the codesign process [11, 12, 13]. Understanding these barriers, alongside enablers, is essential for fostering inclusive environments where young people can meaningfully contribute to research.

Despite growing interest in Codesign across disciplines, the field still grapples with conceptual ambiguity. Our previous research (Authors, forthcoming) highlighted the lack of definitional clarity in how codesign is described and operationalised, often conflated with a broad spectrum of participatory approaches. As Voorberg et al. [14] note, when codesign lacks specificity, it risks being valued for its rhetoric rather than its outcomes. Conceptual clarity is therefore critical for effective implementation and evaluation.

Existing reviews offer important insights. Boswell and Woods [15] examined facilitators and barriers to coproduction with young people in health, education, and social care, emphasising the importance of power‐sharing, flexible methodologies, and sustained commitment. Greenhalgh et al. [2] found that community co‐creation leads to more relevant and sustainable health interventions. Checkoway and Richards‐Schuster [6] argued that youth involvement enhances both data relevance and community capacity. Fleming and Boeck [5] stressed the need for ethical and practical considerations to ensure meaningful participation. These studies, while valuable, often approach youth engagement from broader or sector‐specific perspectives.

McCabe et al. (2023) and Bailey et al. (2024) further illuminate barriers and enablers to youth engagement, particularly in mental health research. Their findings emphasise early and sustained involvement, supportive relationships, and organisational readiness, as well as challenges like unclear roles and resource limitations. However, these reviews focus on youth engagement more generally, rather than codesign as a distinct methodology.

This scoping review builds on this foundation by critically examining how codesign is conceptualised and applied across diverse disciplines and populations, with a specific focus on identifying the barriers and facilitators to its use with young people. In doing so, it offers both conceptual and practical insights to improve future co‐designed research.

By analysing studies that engage young people as co‐designers across various contexts, this review seeks to understand the factors that influence meaningful participation. While other reviews have highlighted elements of youth engagement, our work contributes a distinct lens: one that considers not only what enables or obstructs codesign, but also how the concept itself is mobilised. This includes a focus on theoretical underpinnings and the diversity of populations involved, aiming to clarify inconsistencies and gaps in the literature.

Given the rise in popularity of codesign and the varied ways it is interpreted and implemented, a systematic mapping of its usage with young people is timely. This review aims to provide a nuanced understanding of both methodological and practical considerations to support researchers in conducting inclusive and impactful co‐designed research.

## 2 Methods

A scoping review approach was used to systematically identify and map the breadth of available research using codesign methods and identify key barriers and facilitators related to co‐designed research with young people [16]. This scoping review was conducted in accordance with the JBI (formerly known as Joanna Briggs Institute) methodology for scoping reviews [17] and in accordance with the Preferred Reporting Items for Systematic Reviews and Meta‐Analyses extension for Scoping Review (PRISMA‐ScR) [18]. An unpublished protocol is available from the authors upon request.

### 2.1 Search Strategy

We searched ProQuest, Scopus, Informit, and Science Direct for English‐language, peer‐reviewed publications from January 2003 to August 2023. This time frame reflects the period in which codesign became more prominent in scholarly literature, especially as participatory approaches gained traction across disciplines. The search strategy, developed in consultation with a research librarian, is detailed in Appendix 1.

Our search addressed a broader research question on how codesign is conceptualised in studies with young people (Authors, forthcoming). This paper presents a secondary analysis of those included studies, focusing specifically on barriers and facilitators discussed within them.

Database selection was informed by the interdisciplinary nature of codesign and the research team's expertise in health, education, social sciences, and design. While additional databases such as CINAHL, ERIC, or PsycINFO may have yielded further studies, our selection reflects a pragmatic balance between comprehensiveness and manageability.

### 2.2 Eligibility Criteria

#### 2.2.1 Participants

We included studies involving young people aged 15–24 years, aligned with definitions by the WHO [19] and the International Labour Organization [20]. We also included studies with broader or overlapping age ranges, provided they encompassed this core group.

#### 2.2.2 Concept

We included studies that reported on codesign or related methods—such as participatory research, coproduction, or co‐research—and identified barriers or facilitators to undertaking these methods with young people. Several included studies used elements of patient and public involvement (PPI) along with codesign. The overlap between these approaches reflects the fluidity in how participatory methods are described and enacted in research with young people. Studies that used collaborative methods solely for user testing or feedback, or those that collected youth perspectives without their formal involvement in research processes (e.g., interviews, surveys, focus groups), were excluded.

#### 2.2.3 Context

We considered studies conducted in health, education, disability, public policy, and related social sciences. Only peer‐reviewed articles published in English were included. Grey literature was excluded to maintain consistency in methodological quality and analytical depth.

#### 2.2.4 Types of Sources

We included empirical studies, reviews, evaluations, and methodological papers using qualitative, quantitative, or mixed methods. Unpublished material, grey literature, and laws and regulations were not included.

### 2.3 Study Selection

Search results were managed in Covidence [21]. After duplicate removal, titles and abstracts were screened independently by two reviewers (BL and MS). Disagreements were resolved by including the publication. Full texts were then independently assessed against inclusion criteria by two reviewers (BL and MS) using a predefined inclusion and exclusion criteria, with conflicts resolved through discussion with a third reviewer (HD).

### 2.4 Data Extraction

Data were extracted by two independent reviewers using a pre‐piloted tool within Covidence. A total of seven reviewers contributed to the extraction process (BL, MS, HD, BH, ZA, JB, AK). Extracted data included publication year, country, age group, codesign terminology, study aims, design, theoretical frameworks, participant roles, activities, ethical considerations, and reported barriers and facilitators. Discrepancies were resolved through discussion (BL and MS).

### 2.5 Data Analysis and Synthesis

A qualitative approach was used to synthesise findings, guided by JBI methodology and an inductive content analysis framework [22, 23]. Data were initially organised in Microsoft Word and Excel, with visual mapping conducted in Miro to identify patterns and conceptual groupings.

Researchers (BL and MS) immersed themselves in the included studies, engaging in an iterative process of coding and thematic analysis [22, 23]. This involved returning to the original texts to clarify meaning, refine coding, and identify recurring themes, shared concepts, and overlapping issues. Initial codes were grouped into provisional categories that captured distinct dimensions of co‐designed research with young people.

Through ongoing analysis and discussion, these categories evolved into six conceptual domains—resources, communication, process, agency, investment, and relationships—that represented both the barriers and facilitators to youth engagement. These domains provided a structured yet flexible framework for interpreting the complex, often interrelated, findings across the data set.

## 3 Results

### 3.1 Study Characteristics

**Figure 1:**
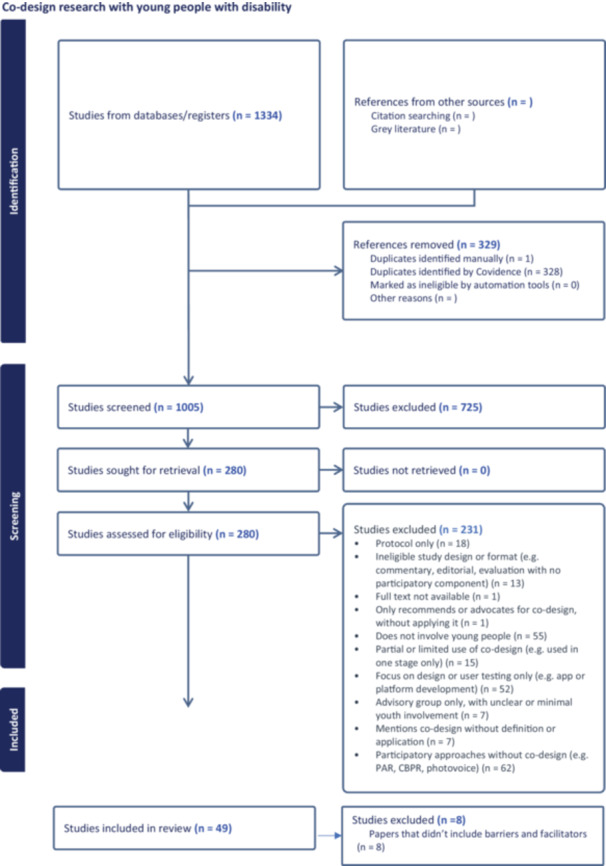
PRISMA_ScR diagram of study selection process.

Forty‐one studies from the 49 studies included in the broader review had information on barriers and/or facilitators to collaborative research and were included in this sub‐question on barriers and facilitators. The flow of studies into the review is shown in Figure 1.

For the 41 publications included in this review, 18 studies were undertaken in the United Kingdom (UK) [24, 25, 26, 27, 28, 29, 30, 31, 32, 33, 34, 35, 36, 37, 38, 39, 40, 41], seven in Australia [42, 43, 44, 45, 46, 47, 48], six in Canada [49, 50, 51, 52, 53, 54], and two in the United States [55, 56]. One study was undertaken in Northern Ireland [57], one in South Africa [58], one in the UK and New Zealand [59], and one across the UK, India, Pakistan, Turkey, Kenya, South Africa, Brazil, and Portugal [60]. Twenty‐nine of the studies used qualitative methods (e.g., focus groups and interviews) [24, 25, 26, 28, 30, 31, 32, 33, 34, 36, 37, 39, 40, 41, 45, 46, 47, 48, 49, 51, 52, 53, 54, 55, 56, 57, 58, 59, 60] and three used mixed methods [35, 42, 44]. Three studies were systematic reviews [15, 61, 62], two were scoping reviews [29, 63], two were text and opinion [27, 50], and two combined qualitative methods with text and opinion [38, 43].

The included age range varied somewhat across studies, with most studies including individuals between the ages of 14–29 years. However, one review included individuals as young as 10 years (range 10–24 years) [63] and one study included participants up to age 26 years (range 14–36 years) [38]. Some studies did not define their age range, instead referring to “young people” [35, 45, 46, 49, 50] or ‘children and adolescents.’ [62]

Most studies discussed both barriers and facilitators to codesign with young people [15, 24, 25, 27, 28, 29, 30, 31, 32, 33, 34, 36, 37, 38, 39, 40, 41, 43, 47, 49, 50, 51, 53, 54, 55, 56, 57, 58, 59, 60, 61, 63], however three studies discussed only barriers [26, 44, 46] and six discussed only facilitators [35, 42, 45, 48, 52, 62].

The studies included a variety of populations. Eleven studies focused on young people with lived experience of mental ill‐health and/or mental health conditions [24, 29, 30, 32, 34, 35, 36, 44, 51, 53, 60] and one study recruited young people with lived experience of self‐harm or suicide of self, friends, or family [59]. Ten studies included young people with disabilities [15, 25, 28, 37, 38, 40, 41, 45, 50, 57], including young people with intellectual disabilities [57], young people with learning disabilities [37, 50], and young people with life‐limiting and life‐threatening impairments [40, 41]. Two studies included Aboriginal and Torres Strait Islander young people in Australia [42, 48] and one study included First Nations young people in Canada [52]. Two studies included young people with experiences of providing care and/or who identified as LGBTQ+ [31, 43]. One study focused on young people who were gang‐affiliated [33].

Included studies addressed a variety of topics, with many addressing mental health [26, 34, 35, 36, 39, 42, 48, 50, 51, 52, 53, 61, 63], including mental health during the COVID‐19 pandemic [35], mental health services [36, 42, 48], mental health care research [61, 63], and topics relating to emotional health [39], self‐management of mental health conditions [51], and mental health care transitions [53]. Studies also addressed sensitive topics such as help‐seeking, emotional abuse and neglect [24], intimate partner violence [58] and family violence [45], and health inequities and racism [56]. Education, health, and care plans [25], transitions in schooling and services [28], supported internship programs [37] and lives and aspirations [40] were topics examined among young people with disabilities and health conditions. Some studies focused on collaborative methods themselves [15, 27, 29, 62], while others aimed to evaluate programs and collaborative approaches [44, 49, 54].

Supporting Information S1: Table S1 provides an overview of included studies, focusing on the barriers and facilitators to engaging youth in participatory research.

### 3.2 Barriers and Facilitators

In our review of the barriers and facilitators influencing co‐designed research with young people, data were most frequently coded to six interconnected domains: resources, communication, process, agency, investment, and relationships. As shown in Table 1, resources (*n* = 25) refer to assets like time, money, and knowledge that support the achievement of project goals [24, 26, 28, 29, 30, 32, 36, 37, 41, 43, 44, 45, 46, 47, 49, 51, 52, 53, 55, 56, 57, 59, 60, 61, 63], while communication (*n* = 20) involves the exchange of information through various channels that foster clarity and collaboration [15, 24, 25, 27, 31, 32, 34, 36, 38, 40, 42, 48, 49, 51, 52, 53, 54, 59, 60, 61]. Processes (*n* = 32) encompass the planning and execution of steps required to achieve specific outcomes, ensuring that efforts are organized and purposeful [15, 24, 25, 26, 27, 29, 30, 31, 32, 34, 35, 36, 37, 38, 39, 41, 42, 43, 46, 47, 49, 50, 51, 52, 54, 55, 56, 57, 58, 61, 62, 63]. Agency (*n* = 22) reflects the ability of participants to influence or control decisions and resources [15, 24, 25, 26, 28, 31, 33, 34, 35, 39, 41, 48, 49, 50, 51, 52, 53, 54, 55, 58, 61, 63], while investment (*n* = 27) refers to the strategic allocation of those resources with the expectation of future benefits, such as capacity‐building [15, 25, 28, 29, 31, 32, 33, 34, 35, 40, 41, 43, 45, 46, 47, 49, 50, 51, 52, 53, 54, 55, 56, 58, 60, 61, 63]. Finally, relationships (*n* = 17) are the connections between individuals or groups, shaped by communication, shared experiences, and mutual influence [15, 24, 31, 33, 34, 36, 37, 41, 48, 51, 52, 53, 54, 55, 58, 61, 63]. Each domain acts as both an enabler and a potential barrier, reflecting the complex dynamics that influence youth participation in co‐designed research. For readers seeking further depth, Appendix Table A1 is an extended version, which includes our full synthesis.

**Table 1:** Summary of domains of barriers and facilitators in co‐designed research with young people.

Domain	Facilitators	Barriers
Resources (*n* = 25) *Assets such as time, funding, staff, knowledge, and infrastructure to support engagement*.	Sustainable funding, skilled staff, diverse engagement methods, use of existing networks, youth co‐facilitation, and role‐based support.	Limited funding/staffing, time constraints, high codesign demands, poor access and logistics, and lack of training or local support.
Communication (*n* = 20) *Exchange of information through inclusive, accessible, and engaging channels*	Transparent and accessible communication, tailored materials, digital and in‐person adaptability, valuing feedback, and critical engagement.	Jargon‐heavy language, digital exclusion, low engagement in online settings, and communication gaps impacting retention.
Process (*n* = 32) *Planning, implementation, and evaluation procedures that structure participation*.	Clear roles, flexible methods, codesign, safe and inclusive spaces, small groups, defined decision‐making, and regular quality assessment.	Tokenism, vague roles, ethical concerns, poor remuneration, unsuitable best practices, and inconsistent engagement frameworks.
Agency (*n* = 22) *Youth capacity to act, decide, and shape research processes*.	Respect for youth agency, power‐sharing, lived experience integration, youth‐led training, and ownership of data and roles.	Stigma, dropouts, unclear expectations, wellness fluctuations, exclusion, and conflicts during data collection.
Investment (*n* = 27) *Time, effort, and leadership commitment to embedding inclusive practice*.	Early leadership buy‐in, emotional and physical accessibility, transparent methods, training, flexibility, and inclusive codesign practices.	Limited diversity, adult‐youth power gaps, disagreement, marginalisation, poor training, and misunderstanding of stakeholder needs.
Relationships (*n* = 17) *Trust, mutual respect, and interpersonal connection among participants*.	Trust‐building, intersectional inclusion, shared decision‐making, mentoring, cross‐sector collaboration, and recognition of youth contributions.	Mismatched groups, lack of trust or confidence, discomfort with sensitive topics, and slow relational development.

## 4 Resources

Resources emerged as both essential enablers and significant barriers. Several studies emphasized the importance of financial support, logistical planning, and accessible environments. For example, Canas et al. [49] noted that mentorship, financial support, and appropriate tools facilitated youth participation, while Fox et al. [43] described how meeting young participants in familiar spaces fostered trust and engagement. However, resource scarcity was also a recurring barrier. Lee et al. [44] reported that limited funding and time constraints disrupted participation, and Mawn et al. [26] highlighted the lack of dedicated funding streams as a threat to sustainable engagement.

### 4.1 Communication

Clear and accessible communication was identified as a key facilitator for motivating and engaging young participants. Dewa et al. [34] highlighted the value of using anonymous feedback mechanisms and accessible tools, such as Padlet, to create safe spaces for youth to share openly. On the other hand, communication breakdowns created challenges. Spuerck et al. [60] found that inadequate communication between youth and adult stakeholders led to feelings of exclusion, limiting engagement.

### 4.2 Process

The structure and processes of co‐designed projects significantly impacted the effectiveness of participation. Studies emphasized the importance of clear role definitions, transparent decision‐making, and youth‐friendly methods. Brady and Franklin described how involving young people in decision‐making fostered a sense of ownership and confidence [25]. However, overly complex processes posed challenges. Dunn reported that rigid governance structures delayed recruitment and limited youth involvement, demonstrating how inflexible processes can act as barriers [36].

### 4.3 Agency

Agency, or the ability of young participants to meaningfully influence projects, was essential to effective codesign. Boswell and Woods showed that power‐sharing from the start of the project promoted meaningful involvement [15]. However, power imbalances remained a persistent barrier. Mannell et al. [58] found that disagreements between youth participants and researchers over data interpretation reflected ongoing struggles with power dynamics, which complicated collaboration. Fluctuations in wellness and personal circumstances also limited agency, as seen in Lapadat et al. [51], where some participants dropped out due to challenges in balancing commitments.

### 4.4 Investment

Sustained investment in time, effort, and resources was critical to building capacity among youth participants. Taylor et al. [31] highlighted that significant staff time invested in relationship‐building strengthened the codesign process. However, Mulvale et al. [53] noted the difficulty of sustaining youth involvement, especially when participants faced competing demands on their time. A lack of organizational investment often limited the depth of youth participation.

### 4.5 Relationships

Trust‐based relationships formed the foundation of successful co‐designed projects. Liebenberg et al. [52] emphasized how partnerships with community organizations helped build trust and facilitated deeper engagement. However, insufficient relationship‐building efforts or unchecked power imbalances undermined collaboration. Mawn et al. [26] noted that without continuous relationship‐building activities, participants often disengaged, leading to disruptions in the codesign process.

These results underscore the importance of aligning research practices with the needs, experiences, and capacities of young people, ensuring their participation is not tokenistic, but is instead transformative. The reviewed publications reveal that participatory research with youth offers numerous benefits, from empowering young people to enhancing the relevance of research outcomes. However, meaningful engagement requires intentional efforts to address power dynamics, logistical challenges, and funding limitations. The facilitators identified—such as relationship‐building, flexible methodologies, and shared decision‐making—are pivotal to overcoming these barriers. Moving forward, research efforts must focus on creating sustainable models that foster trust and provide the necessary resources for youth engagement to thrive.

## 5 Discussion

This review identified barriers and facilitators to engaging with young people in co‐designed research, synthesizing the factors which acted as facilitators and barriers across six key domains. We found that the success of codesign projects hinges on effectively addressing multiple interconnected domains, where barriers are also often simultaneously enablers to good codesign practices. These domains—resources, communication, process, agency, investment, and relationships—form a complex, interdependent framework. For a codesign project to reach its full potential, all domains must be addressed holistically, acknowledging that their interconnectedness drives the codesign process forward.

### 5.1 Enabling and Limiting Participation

Resources often form the foundation for project feasibility. Resources such as time, money, knowledge and tools can support effective codesign, but their availability often determines the feasibility of participation. As seen in Canas et al. [49], financial support and mentorship encouraged youth engagement by reducing logistical barriers [49]. However, the absence of sufficient resources, as highlighted by Lee et al. [44], constrained their project's ability to engage youth fully, illustrating the duality of this domain. Investment in accessible environments and diverse methods may help address participation barriers, but resource limitations frustrate capacity‐building efforts, particularly in marginalized communities.

The use of diverse, accessible methods can help reduce logistical burdens and potentially create more inclusive environments. For example, Robinson et al. [45] illustrates how concentrated mentoring and supervision ensured that young people with disabilities could meaningfully participate. Similarly, Wright et al. [48] reported that the use of culturally appropriate engagement methods, like On Country activities, enabled deeper participation among Aboriginal youth, showing how tailored resources can enhance inclusivity. However, the absence of accessible transport and support staff, as noted by Rome et al. [28], suggest that logistical challenges remain a key obstacle. Resource shortages—whether in terms of time, funding, or staffing—can become significant barriers, inhibiting genuine participation. Insufficient funding, inadequate staffing, and limited timelines frustrate capacity building and mentorship, which are essential for the active engagement of co‐researchers. Thus, while resources may enable participation in some settings, their scarcity can may limit it in others.

### 5.2 Communication as Key to Inclusion

Communication plays an important role in shaping collaborative dynamics. When clear and inclusive, it can enhance project clarity and support engagement. Tools like anonymous feedback mechanisms used by Dewa et al. [34] exemplify how effective communication creates safe spaces for open expression. However, miscommunication or a lack of appropriate tools, as seen in Spuerck et al. [60], can alienate young participants and disrupt engagement. Goodley et al. [38] demonstrated how early sharing of conceptual models with young participants promoted coproduction by making the research process transparent. In contrast, Knowles et al. [59] found that interactive debates—although valuable—presented challenges in balancing diverse perspectives.

Maintaining adaptive, youth‐friendly communication strategies appears important for sustaining engagement. However, challenges such as inaccessible language or ineffective use of digital platforms can marginalise participants or limit shared understanding. In codesign contexts that depend on the continuous exchange of ideas, communication—when mismanaged—may disrupt participation and undermine collaborative intent.

### 5.3 Balancing Structure and Flexibility

Co‐designed projects appear to benefit from structured but adaptable processes. Davison et al. [57] emphasized that processes need to be tailored to participants, such as using pictorial prompts for youth with intellectual disabilities, ensuring their involvement is meaningful. Clear role definitions, transparent decision‐making processes, and tailored methods to empower young participants, also offer structure and youth ownership over the project. However, the complexity of these processes can also slow progress and increase the risk of tokenistic involvement, where young people are superficially included without meaningful engagement. Time‐consuming procedures, lack of robust ethical frameworks, and power imbalances further compound these challenges, demonstrating that while a clear process structure is crucial, its rigidity can serve as a barrier if not thoughtfully designed.

While structured processes help create transparency and clarity, they must remain flexible to prevent tokenism. Giving youth ownership over decisions can enhance their confidence and contribution to policy changes [25]. However, rigid governance structures, as noted by Dunn, delayed recruitment and reduced the project's effectiveness [36]. A well‐structured process empowers participants, but it must also be adaptable to accommodate their evolving needs. Taggart et al. [30] highlights the difficulty of mental health needs, as complex processes risk becoming overwhelming, especially for participants experiencing mental health challenges. This demonstrates the need for processes that are both structured and sensitive to participants' well‐being.

### 5.4 Empowerment Through Power‐Sharing

While empowerment is frequently cited in the literature as a desirable outcome, our review highlights the need for empirical evaluation of its implementation in codesign contexts. Agency is often linked to the ability of young people to shape project directions based on their experiences. While early power‐sharing may enhance engagement [15], unresolved imbalances—such as those documented by Mannell et al. [58]—can limit collaboration. Empowering youth requires more than tokenistic involvement; it demands consistent efforts to balance power and support participants through personal challenges, as seen in Lapadat et al. [51]

Participatory methods have the potential to challenge conventional power structures [43], but enabling youth agency in practice is not always straightforward. Adults may be reluctant to relinquish control [46], and the emotional labour of participation can become burdensome for young people, leading to disengagement. These dynamics point to the importance of clear roles, sustained support, and shared expectations to strengthen agency in ways that are contextually appropriate.

This is a persistent tension in collaborative research between facilitating youth agency and maintaining project oversight. There may also be an emotional burden to the intensity or responsibility of power sharing, and this may cause youth engagement to drop‐off, which can disrupt continuity and impact data collection. This highlights the importance of clear roles and expectations to sustain agency. Thus, while agency facilitates ownership, its fragility reveals its dependence on sustained support and balanced power dynamics.

### 5.5 Sustaining Engagement

Investment—of time, energy, and resources—has been linked in several studies to longer‐term engagement, particularly with marginalised groups. Taylor et al. [31] reported that relationship‐building contributed to sustained youth participation, while Woods‐Jaeger et al. [56] noted that unfamiliarity between participants and researchers hindered collaboration. Engaging facilitators closer in age to participants may also help build trust [32]. Underscoring the importance of early investment in relationship‐building activities. Employing facilitators of a similar age can also improve youth engagement and trust [32]. The level of sustained investment, or “political will” from project leaders plays a big role in the ongoing success of a codesign project. Without adequate investment, co‐designed projects risk becoming short‐term endeavours that fail to achieve meaningful impact.

The role of leadership and institutional support—or lack thereof—can influence whether codesign projects maintain momentum or remain short‐lived. When investment is limited, youth involvement may become episodic or superficial, affecting both outcomes and the integrity of the process.

### 5.6 The Social Fabric of Codesign

Relationships shaped by trust, respect, and reciprocity have emerged as central to successful codesign in many studies. Partnerships with community organisations, such as those described by Liebenberg et al. [52], have been shown to support deeper youth engagement. However, maintaining strong relationships requires ongoing effort, and disruptions—such as staff turnover or insufficient time allocation—can lead to disengagement [26].

Building meaningful relationships requires ongoing effort, particularly in contexts where power imbalances may undermine trust. Lapadat et al. [51] underscored the value of regular check‐ins, shared meals, and mutual respect to foster trust between youth participants and researchers.

The relational aspect of codesign is particularly important when working with geographically dispersed groups or in digital contexts. Ho et al. [64], for example, found that online mental health interventions were often met with scepticism due to concerns about trust and security. These findings suggest that fostering meaningful relationships—whether in‐person or online—may benefit from integrated, user‐centred design strategies and attention to continuity.

Strong relationships can support shared leadership and build project capacity, but their fragility underscores the need for ongoing care, particularly when power imbalances persist or relational labour is undervalued. The influence of interpersonal dynamics on project outcomes further illustrates the interdependence of the domains discussed.

In sum, there is not a one‐size‐fits‐all model for codesign, but rather, all projects rely on the interconnection of these domains. Resources, communication, process, agency, investment, and relationships each serve as both enablers and barriers, depending on how they are managed. A project that successfully integrates these domains holistically, recognizing their cross‐connections and interdependence, is far more likely to achieve meaningful and sustained collaboration.

### 5.7 Limitations of Study

This study has several limitations. First, relevant research may have been excluded due to the omission of grey literature. Additionally, as the included studies were drawn from specific disciplines and contexts, the generalisability of the findings may be limited. Further investigation across a broader range of fields is needed before drawing firm conclusions about the barriers and facilitators to co‐designed research with young people.

We also acknowledge the absence of direct involvement of young people in this review as a limitation. While primary engagement was not required for the scoping methodology, future research would benefit from participatory approaches that centre young people in the interpretation of findings and development of conceptual frameworks.

Another limitation relates to the nature of the data: our findings are based on both young people's accounts within the original publications and the interpretations of the researchers who conducted those studies. This reliance on secondary data introduces the potential for interpretive bias, which may affect how youth perspectives are represented and, in turn, limit the applicability of the conclusions.

Finally, although our initial search did not specifically target studies focused on barriers and facilitators, we took a fine‐toothed comb to the papers included in this study, which enabled us to capture the nuances to barriers and facilitators in co‐designed research with young people. However, In this scoping review, it was essential to examine all relevant papers for potential insights on barriers and facilitators related to co‐designed research with young people, rather than limiting the focus to studies explicitly centered on these themes. This broader approach allowed for a more comprehensive understanding of how various aspects of codesign processes influence outcomes and interactions with young participants.

## 6 Conclusion

This scoping review identified methodological and practical consideration for researchers looking to use codesign with young people in health research, service design, re‐design or quality improvement initiatives. The key findings relate to improving the experience for young people when engaging in codesign research and to the development of codesign methods themselves. We found that the facilitators and barriers identified are not isolated elements but rather interdependent factors that collectively shape the success of projects co‐designed with young people. Each project is unique, with its own set of complexities and challenges, necessitating careful, context‐specific planning.

These findings support the need for further research into the transferability of flexible and adaptive codesign tools with young people, particularly if codesign is to become a best practice method. Nevertheless, across any project, the effective alignment of scope and resources is essential, as it ensures that projects are both feasible and sustainable.

Additionally, sustained leadership and commitment are critical in maintaining momentum and fostering long‐term success. At the core of any project, however, must be the empowerment of young people. This requires not only the provision of adequate support and resources but also a genuine commitment to involving youth as active co‐creators. Their insights and experiences should guide the project's direction and outcomes, reinforcing a participatory approach that honours their agency. Ultimately, the careful orchestration of these factors is fundamental to overcoming barriers and enabling meaningful, impactful youth engagement.

## Author Contributions

**Briony Lipton:** conceptualization, investigation, writing – original draft, methodology, writing – review and editing, project administration, formal analysis, visualization, data curation, validation. **Helen Dickinson:** conceptualization, writing – review and editing, formal analysis, investigation. **Jodie Bailie:** writing – review and editing, formal analysis, data curation. **Belinda Hewitt:** conceptualization, formal analysis. **Anne Kavanagh:** conceptualization, formal analysis. **Zoe Aitken:** conceptualization, formal analysis. **Marissa Shields:** conceptualization, investigation, writing – original draft, methodology, writing – review and editing, formal analysis, data curation, visualization, validation.

## Ethics Statement

The authors have nothing to report.

## Consent

The authors have nothing to report.

## Conflicts of Interest

The authors declare no conflicts of interest.

## Supporting information

Table A1 Scoping review barriers and facilitators.

Table S1 Descriptions of included papers.

Appendix 1 Search strategy 20 8 2024.

## Data Availability

Further information on extracted data is available from the corresponding author on reasonable request.
