# Supplementary material for: Collaborating With Young People: Identifying the Barriers and Facilitators in Co‐Designed Research
Source: Health Expect. 2025 May 27;28(3):e70308. doi: 10.1111/hex.70308 (PMC12117193; doi:10.1111/hex.70308)
Supplement: Supplementary file 1 — Table A1 Scoping review barriers and facilitators. [file HEX-28-e70308-s001.pdf]

TableA1: Detailed domains of barriers and facilitators identified in co-designed research with young people

Domain		
	**Facilitators**	**Barriers**
**Resources** (n=25)<br>*Assets or materials, such as time, money, knowledge, and tools, that are available and utilized to achieve specific goals or support activities.*	• **Ensure continuous funding and resource availability** to support the project's sustainability and success.<br>• **Respect the experiences and knowledge of those involved** to validate contributions and enhance collaboration.<br>• **Appoint a dedicated youth engagement coordinator** to focus on fostering effective participation and support for young people.<br>• **Involve young people as co-facilitators** to empower them and enhance their leadership skills.<br>• **Provide different roles, opportunities and additional support** based on strengths and interests of young people.<br>• **Assign dedicated staff for support** to provide guidance and assistance throughout the research process.<br>• **Embed longer timelines for capacity building into the project** to allow for meaningful development and growth.<br>• **Maintain consistent involvement of young people (i.e. as co-researchers)** to build continuity and strengthen collaboration.<br>• **Utilize community service providers, networks, and stakeholders** to enhance the	• **Lack of resources, including time and funding, limits genuine youth participation** by creating obstacles to engagement.<br>• **High costs associated with developing youth culture in research** restrict opportunities for meaningful involvement.<br>• **Inadequate staffing restricts project capacity** by limiting support and resources for participants.<br>• **Significant time investment is required for co-design** which can hinder the project's timeline and progress.<br>• **Insufficient duration and funding limit training and analysis opportunities** for participants and staff.<br>• **Limited resources hinder mentorship and collaboration efforts** by reducing support for relationship building.<br>• **Lack of research skills can pose challenges in data collection** and affect the quality of outcomes.<br>• **External responsibilities, cost constraints, recruitment challenges, and transportation limitations** create barriers to participation.<br>• **Existing barriers with local services limits interest and involvement** and reduces the

	project's reach and effectiveness.<br>• **Tailor diverse, accessible, and flexible methods to the specific needs of the group** to maximize engagement and participation.<br>• **Involve established groups where possible** to leverage familiarity and ease in collaboration.<br>• **Use resources to simplify logistics and reduce the burden on young people** to enhance their participation and focus on the research.	support available for the project.
**Communication** (n=20)<br>*The exchange of information, ideas, and feelings between individuals or groups through various methods, including verbal, non-verbal, written, and digital channels.*	• **Communicate how and why collaborative research methods are used** to ensure transparency and justification for involving young people in the research process.<br>• **Use accessible language and tailored materials** to enhance understanding and engagement among participants.<br>• **Employ youth-friendly communication tools** to increase participation and comfort for young people in the project.<br>• **Implement pre- and post-briefing sessions for large meetings** to ensure clarity and alignment among participants.<br>• **Provide quiet spaces for collaborative work** to foster focused and productive group interactions.<br>• **Ensure strong communication and support** to build trust and encourage ongoing involvement from participants.	• **Lack of communication** negatively impacts decision-making and the effectiveness of meetings.<br>• **Insufficient use of accessible language and layperson-friendly documentation** creates confusion and limits participation.<br>• **Lack of digital skills and access** hinders effective virtual collaboration and engagement.<br>• **Online meetings often lack engagement from young people**, resulting in diminished participation and interest.<br>• **Digital exclusion** restricts access for some participants, limiting their ability to contribute.<br>• **Communication barriers** significantly impact retention and participation.

	• **Foster motivation through open communication** to inspire active participation and commitment to the project.<br>• **Prioritize effective communication, including consistent contact and flexibility across all environments (digital, in-person, and hybrid)** to support sustained engagement and adaptability to participants' needs.<br>• **Clarify project possibilities** to empower participants with a clear understanding of their roles and contributions.<br>• **Respect peer researchers' opinions** to cultivate an inclusive environment where all voices are valued.<br>• **Welcome critical perspectives** to encourage diverse viewpoints and enhance the quality of research outcomes.<br>• **Allocate time for reflection and challenge research epistemologies** to promote deeper understanding and critical thinking among participants.	
**Process** (n=32)<br>*A series of actions, steps, or procedures taken to achieve a particular outcome, often involving planning, implementation, and evaluation.*	• **Provide clear structure and support throughout the research process** to enhance participant confidence and engagement.<br>• **Define roles and responsibilities clearly** to ensure everyone understands their contributions to the project.<br>• **Establish processes for building young people's capacity to participate** to	• **Time-consuming procedures hinder study progress, recruitment, and training** by delaying essential processes.<br>• **Lack of processes can lead to inequities and ethical dilemmas, such as exploitation** by creating inconsistencies in participation.<br>• **Tokenistic involvement limits meaningful participation** by reducing young people's role to mere formality.

	empower them and foster active involvement.<br>• **Create and embed safe spaces and channels for support and inclusion** to promote a welcoming environment for all participants.<br>• **Assign concrete responsibilities for engagement** to encourage accountability and active participation.<br>• **Develop collective principles of work** to guide collaboration and mutual respect among participants.<br>• **Keep group sizes small** to enhance participation and foster meaningful interactions.<br>• **Define decision-making power at each stage** to clarify roles and enhance participant ownership of the process.<br>• **Give young people decision-making power** to empower them and enhance their commitment to the project.<br>• **Sustain trusting relationships through transparency** to build a positive and collaborative atmosphere.<br>• **Involve young people in project design and methods** to ensure their perspectives are reflected in the research.<br>• **Choose study methodologies that support engagement and enhance idea generation** to encourage creative input from participants.<br>• **Offer flexibility in project focus** to adapt	• **Lack of quality assessment tools** undermines efforts to ensure ethical involvement and protect participants' rights.<br>• **Not offering market-level remuneration as fair compensation for young people's contributions** produces inequities.<br>• **Power imbalances and unclear roles pose challenges** by creating confusion and limiting engagement.<br>• **Generalized best practices are difficult to implement** because they don't often consider the diverse needs of all parties.<br>• **Frameworks are needed to guide best practices** and ensure consistent approaches across projects.<br>• **Young people juggle multiple roles, complicating their engagement** and making it challenging to participate fully.

	to the evolving interests and needs of participants.<br>• **Encourage peer collaboration** to foster a sense of community and shared responsibility.<br>• **Use multiple virtual environments** to enhance inclusivity and accommodate diverse preferences.<br>• **Select appropriate study methodologies and adapt methods as needed** to engage young people effectively and stimulate idea generation.<br>• **Engage external consultants for evaluation** to bring in fresh perspectives and ensure quality assessment.	
**Agency** (n=22)<br>*The capacity of an individual, group or organisation to make choices, act independently, and the ability to shape one's actions and decisions, direct others and resources to achieve desired outcomes.*	• **Respect the agency and interests of young people** to empower them and encourage authentic participation.<br>• **Share power and treat youth as equals** to foster a collaborative and inclusive atmosphere.<br>• **Co-create accessible and understandable topic guides** to enhance comprehension and engagement in the research process.<br>• **Acknowledge the possibilities and limitations of collaborations** to set realistic expectations and build trust among participants.<br>• **Implement youth-led training initiatives** to foster agency among groups and individuals.	• **Difficulty managing expectations and sustaining engagement** hinders participants' commitment and project success.<br>• **Lack of diversity and potential exclusivity in co-production** limits representation and the richness of perspectives.<br>• **Recruitment challenges and funding continuity issues** restrict capacities and impede project development.<br>• **Stigma, power imbalances, and location barriers impact participation** by creating obstacles to engagement.<br>• **Fluctuating wellness affects ongoing engagement** and can lead to inconsistent participation.

	• **Ask for and actively listen to feedback** to demonstrate value for participants' perspectives and improve the project.<br>• **Foster a recovery-focused environment** to support well-being and resilience among participants.<br>• **Value the expertise of lived experience** to enrich the research process with authentic insights.<br>• **Encourage interpretive ownership of data** to empower young people in understanding and utilizing their contributions.	• **Unclear expectations and responsibilities impact young people's sense of agency** by creating confusion about their roles.<br>• **Dropouts disrupt project management and data collection** by creating gaps in information and continuity.<br>• **Emotional impacts on co-researchers affect their well-being** and may hinder their ability to contribute effectively.<br>• **Conflicting priorities during data collection** complicate participation and can lead to disengagement.
**Investment** (n=27)<br>*The allocation of resources, such as time, effort, or capital, into activities or assets with the expectation of generating returns or achieving future benefits.*	• **Invest early and continuously from leadership** to establish a strong foundation for the research.<br>• **Prioritize emotional and physical accessibility throughout the research** to ensure all participants can engage fully.<br>• **Be transparent about collaborative methods from the outset** to build trust and clarify expectations.<br>• **Ensure flexible work modes and provide mentoring** to accommodate diverse needs and enhance support for participants.<br>• **Provide training to adult collaborators on working with young people** to facilitate effective communication and collaboration.<br>• **Maintain flexibility with time and location** to accommodate participants' schedules and preferences.	• **Unexpected delays or events may occur** and undermine investment in the project.<br>• **Lack of diversity in representation** limits diverse perspectives and inclusivity in the research.<br>• **Sustaining engagement while balancing diverse perspectives** can create challenges in collaboration.<br>• **Unresolved disagreements between youth researchers and other team members** hinder progress and morale.<br>• **Adult attitudes and assumptions can limit collaboration** by creating power imbalances.<br>• **Marginalization and restricted involvement in decision-making** prevent meaningful contributions from young people.<br>• **Need for reflection on practices and ensuring meaningful contributions** to

	• **Conduct workshops in familiar settings** to create comfort and encourage participation.<br>• **Use adaptable approaches to foster engagement** and respond to the evolving dynamics of the group.<br>• **Leverage technology for communication and leadership** to enhance coordination and connectivity among team members.<br>• **Influence all stages of the study to drive meaningful change** by ensuring active involvement from participants.<br>• **Allocate time for experience mapping and co-designing solutions** to incorporate diverse insights and foster ownership of the outcomes.	improve the quality of collaboration.<br>• **Lack of training on relevant social research models** (e.g., social model of disability, decolonizing models for working with First Nations young people) limits effectiveness.<br>• **Need for training in participatory approaches and incorporating diverse perspectives** for both young people and adults to enhance collaboration.<br>• **Limited understanding of stakeholder and adult viewpoints** can hinder effective communication and alignment.
**Relationships** (n=17)<br>*The connections, associations, or interactions between individuals, groups, or entities, often characterized by shared experiences, communication, and mutual influence.*	• **Build safe and trusting relationships to minimize power imbalances** and create a supportive environment for all participants.<br>• **Recognize and account for intersectional experiences** to ensure inclusivity and understanding among participants.<br>• **Utilize relationships to strengthen research capacity across all participants** by fostering collaboration and knowledge sharing.<br>• **Promote leadership capabilities at all levels** to empower participants and enhance project outcomes.<br>• **Revisit key themes and discuss**	• **Managing diverse attitudes and opinions effectively** can be challenging and may create tension in discussions.<br>• **Ensuring the selection of appropriate participants** is crucial, as mismatched groups can hinder engagement.<br>• **Addressing initial unfamiliarity between youth and academics** may create barriers to effective communication and collaboration.<br>• **Failing to support the development of young participants' confidence** can hinder their engagement and contributions.<br>• **Engaging youth while navigating differing opinions in focus groups and discussions**

	**summaries collaboratively** to ensure shared understanding and collective ownership of the research.<br>• **Collaborate across disciplines and with stakeholders** to enrich the research with diverse perspectives and expertise.<br>• **Give recognition and acknowledgment for the training undertaken by young people for the project** to validate their contributions and enhance motivation.<br>• **Create warm and welcoming environments** to encourage open communication and participation.<br>• **Dedicate time to relationship building** to foster trust and effective collaboration.<br>• **Address differing priorities through shared language** to facilitate understanding and cooperation.<br>• **Balance conflicting priorities effectively** to maintain focus and cohesion within the group.<br>• **Provide mentorship and opportunities for relationship building** (both formal and informal) to enhance support and connection among all parties.	can complicate consensus-building.<br>• **Inadequate time and effort invested in relationship building** limits trust and collaboration among participants.<br>• **Reluctance to discuss sensitive topics** can stifle open communication and hinder the exploration of important issues.<br>• **Slow progress in sharing personal information** can impede relationship development and trust-building.
