# Supplementary material for: Collaborating With Young People: Identifying the Barriers and Facilitators in Co‐Designed Research
Source: Health Expect. 2025 May 27;28(3):e70308. doi: 10.1111/hex.70308 (PMC12117193; doi:10.1111/hex.70308)
Supplement: Supplementary file 2 — Table S1 Descriptions of included papers. [file HEX-28-e70308-s003.pdf]

Table S1: Description of studies included in the review of barriers and facilitators

First author; year; country	Study design; study purpose and focus; participant characteristics	Factors: Facilitators	Factors: Barriers
Ali; 2023 (1)	Scoping review<br><br>Identify approaches to engagement; patient engagement in mental health care research<br><br>Adolescents and youth aged 10-24 years	• Recruiting a diverse participant sample facilitates engagement.<br>• Holding multiple workshops at different sites and in familiar environments supports participation.<br>• Allocating time to build trust and rapport with youth co-researchers fosters meaningful engagement.<br>• Selecting study methodologies that engage young people enhances participation and idea generation.<br>• Employing workshop facilitators and using multiple methods to gather data promotes effective collaboration.	• Recruitment may be challenging, resulting in small numbers of participants and under-representation.<br>• Recruitment and participatory involvement can be barriers (e.g., limited numbers of young people who can participate) or a constraint (e.g., study inclusion criteria is narrow).<br>• Limited number of participants can restrict activities or limit input into processes.<br>• Difficulty managing youth expectations of project outcomes.<br>• Youth participation may be impacted by stigma around mental health, power imbalances between participants, and the location of activities.<br>• Governance may be time-consuming, and time limits may hinder prolonged engagement, impacting study progress.
Bennett; 2022a; UK (2)<br><br>"coproduction to understand"	Qualitative research<br><br>Explore impacts of young co-researchers involvement in research; help-seeking, emotional abuse, and neglect	• Providing a clear structure and consistent support throughout the research process facilitates engagement, especially on sensitive topics.<br>• Leveraging an already established group of young people reduces the need for additional time to develop rapport.<br>• Incorporating flexibility, regular	• The project occurred during the COVID-19 lockdown so had to be adaptable.

	Aged 14-18 years; male, female, and gender non-conforming; some had lived experience of mental health challenges; White British; most attending state-funded schools	engagement, and online tools (e.g., virtual meetings, breakout rooms) enhances research processes, motivation, and capacity building.<br>• Skills development, increased confidence, and the opportunity to have their voices heard empower young co-researchers.<br>• - Minimising one-on-one work between researchers and young people, unless specifically requested, supports comfort and collaboration.<br>• Embedding feedback mechanisms throughout the research process ensures adaptability and responsiveness.<br>• Seeking feedback at the project's start to adapt methodologies and assess capabilities enables early, necessary adjustments.<br>• Creating opportunities for ongoing, reflective feedback through formal, informal, and anonymous methods (e.g., padlet) fosters a safe environment for sharing insights.<br>• Building a safe, respectful environment and maintaining balanced representation between young people and adult researchers supports positive relationships and collaboration.	
Boswell; 2021 (4)	Systematic review<br><br>Investigate facilitators and barriers of co-production of services; co-production methods	• Adults need to be open to learning about young people and their skills and engaging in relationship-building activities such as games.<br>• Relationships need to have a more personal element.<br>• Professionals need support to feel comfortable sharing power.	• Recruitment can be difficult, particularly among marginalized groups. Some young people may be uninterested due to the research design.<br>• Recruitment may be better when young people are involved in the design of the research; adult-led

	Child or young person ≤25 years with special educational needs and disability or ≤18 years without special educational needs and disability	• Power sharing is necessary to involve young people from the beginning of the research.<br>• Support is required from leaders to ensure research goes well and systems need to be reflexive.<br>• Researchers need to consider practicalities such as transport, location, and timing flexibility.<br>• Provide quiet spaces for young people and send research information out in advance.<br>• It is important that young people feel genuinely seen and heard. Remuneration was provided in some projects.<br>• Early involvement in practice and research is key to more active participation. Clear co-production aims from the outset may facilitate this.	agendas may be barriers.<br>• Professionals may view young people as incapable of participation and may marginalize their understanding of their own situation.<br>• Practitioners may not seek feedback from young people about practice and may not involve them in decision-making.<br>• Early investment is required from leaders to make co-production work. It can be difficult to get wider teams invested in co-production.
Brady; 2019; UK (5)	Qualitative<br><br>Describe co-led research process; defining quality and rights-based Education, Health, and Care Plans<br><br>Aged 16-23 years; all with disabilities	• Involving young people in decision-making throughout the process motivated them and helped them understand how their research could impact on policy and practice.<br>• Through participation, young people gained skills, confidence, friendships, and felt proud of themselves.<br>• The researchers created a document about the young people involved in the research to share what they needed to know to keep them safe while undertaking the research, including information on likes and dislikes and accessibility. Information was only asked for on a need-to-know basis.<br>• Researchers were careful to under-promise and over-deliver and were	• Found that other staff did not have good training or understanding of the social model of disability, leading to outdated views and questions.<br>• Young people were emotionally impacted by witnessing and hearing about injustices to others' lives. Researchers raised concerns regarding the impact of the negative research evidence on young people's wellbeing and whether it was right to expose young people to this.

		clear about what was possible over the course of the project; this aided relationship building.	
Canas; 2019; Canada (6)	Qualitative<br><br>Evaluate youth engagement; participatory evaluation of engagement<br><br>Young people with prior experience in organizations (age not reported)	• Mentorship, skills building, financial and reputational resources, and clear communication and expectations generated effective engagement activities.<br>• Mentorship provided the flexibility to talk, brief and debrief, and build knowledge.<br>• It was important to be flexible and accommodate the wellness of individuals.<br>• Providing effective scaffolding and support techniques in developing events allowed young people with different levels of skills to participate.<br>• Engaging an external consultant to run the evaluation may have made it easier for participants to have an honest discussion.	• Limited training opportunities for young people restricted their ability to effectively contribute to the strategic direction of the program.<br>• Insufficient avenues for young people to provide direct input into the program's vision and strategic development hindered meaningful engagement.<br>• A lack of training for adult board members in participatory approaches reduced the effectiveness and sustainability of youth engagement efforts.<br>• Keeping youth engaged can be challenging as it is difficult to sustain everyone's interest and adapt to everyone's schedules.<br>• Online meetings are not as engaging.<br>• Fluctuations in young people's wellness (physical, mental, and financial) are a determinant of their engagement. It can be difficult for youth to prioritize these kind of roles.<br>• Discussions may move too quickly and young people may need more time and clarification to fully participate.<br>• There are challenges with communication between young people, the board, and the rest of the youth council. Young people felt that there was less communication with and impact upon Board decisions and

			program direction.<br>• Young people need market-level remuneration for their involvement. Volunteer activities are unsustainable.<br>• There was limited time and resources to implement mentorship practice with young people.
Culbong; 2022; Australia (7)	Mixed methods<br><br>Improve cross-sector collaboration; mental health services for Aboriginal and Torres Strait Islander young people<br><br>Aboriginal or Torres Strait Islander young people aged 16-25 years	• Using appropriate methods (e.g., yarning) established informal boundaries and protocols of the research relationship.<br>• Using yarning methods allowed space for the research to be flexible and responsive.<br>• Sharing stories was powerful and helped to develop trusting relationships to ensure collaborative processes.	
Davison; 2022; Northern Ireland (8)	Qualitative<br><br>Provide guidance for participatory research methods; adapting self-report measures of wellbeing<br><br>Aged 11-17 years, male and female; all with intellectual disabilities	• Using pictorial prompts and visual imagery helped young people with intellectual disabilities participate.<br>• Structuring the workshops to suit the needs of young people with intellectual disability was necessary (e.g., presenting items one at a time with a clear structure).	• Young people with intellectual disabilities had difficulty with abstract concepts and working memory demands. This required study material to be adapted.
Dewa; 2021a; UK (9)	Qualitative<br><br>Describe co-production approach; mental	• The co-researchers created the topic guide, which was written in a way that young people could understand and was appropriate for the audience. This helped interviews feel like conversations and helped the young	• The personal experience of young people sometimes impacted interviews, i.e., they may use leading questions because of their mutual mental health experience. This could potentially affect the integrity of the

“Reflections”	health<br><br>Aged 18-25 years; male and female; White British, British Asian, and Black British ethnicities; lived experience of mental health difficulties	people use appropriate probing questions.<br>• Reflection and feedback sessions after interviews helped young people improve their interview skills.<br>• Creating and maintaining relationships was vital throughout the project.<br>• Involving the co-researchers throughout the whole project and not intermittently led to meaningful involvement and true co-production.	data.<br>• Several young people dropped out of the project which impacted project management, and data collection, analysis, and dissemination.<br>• There were sometimes conflicting priorities due to the different experiences and backgrounds of the project team.<br>• Balancing power was difficult as the lead researcher had to maintain overall responsibility for the project including organizing activities, resources, planning and logistics, meetings and events, and was responsible for the co-researchers safety, finances, and potential errors.<br>• Building trust and relationships and undertaking collective decision-making took more time, budget, and dedication than predicted.<br>• The young people felt there was not enough time taken to explaining aspects of the project at the beginning.
Dewa; 2021b; UK (10)<br><br>“CCoPEY”	Mixed methods<br><br>Examine mental health and coping during COVID-19 lockdown; mental health impacts of COVID-19<br><br>Age not reported; co-researchers had experiences of mental health difficulties	• Each co-researcher was given a university email address and contingent worker status to ensure that all co-researchers had access to all project communications and helped to ensure equal power between researchers and co-researchers.<br>• All co-researchers received training (e.g., mental health first aid course).<br>• Debriefs were conducted when necessary and two psychiatrists were on-call during the interviews in case	

		further support was needed.	
Dunn; 2017; UK (11)	Qualitative<br><br>Co-produce a Transition Preparation Program; mental health services<br><br>Aged 16-22 years; male, female, and gender-fluid; had transitioned or were approaching transition from Child and Adolescent Mental Health Services and were involved in participation networks; experiencing a variety of mental health and other conditions	• Creating a positive and interactive environment facilitated open conversation.<br>• Young people enjoyed the creative methods that were used and the variety of activities	• Governance procedures were more time-consuming than anticipated which delayed recruitment, resulted in a small sample, and insufficient time to organize and train young people for some of the planned co-research activities.<br>• The 12-month duration of the study was insufficient for participants to receive training and carry out parts of the planned analysis.
Edwards; 2022; UK (12)	Qualitative<br><br>Explore experiences of special educational needs and disabilities; supported internship programs<br><br>Aged 18-25 years; male and female; had learning disabilities and some other disabilities; members of ethnic minority groups; have education, health and care plan and be enrolled in a	• Revisiting themes and discussing summaries may have helped young people feel their views were taken into account.<br>• Involving co-researchers in generating interview questions facilitated power sharing.<br>• The project had a degree of flexibility which allowed co-researchers to share what the most important issues to them were.<br>• Giving the co-researchers the opportunity to choose the methods worked well.	• Research was time consuming, requiring a number of sessions and placing a time burden on the co-researchers.

	supported internship programme		
Fox; 2019; Australia (13)	Qualitative, text and opinion<br><br>Examine critical methodologies; experiences of young primary carers in rural Australia; experiences of LGBTQI young people in rural Australia<br><br>Study 1: 12-17 years; male and female; primary carers in rural Australia<br><br>Study 2: young people identifying as LGBTQI and living in rural Australia	• Meeting in an environment familiar to young people corrects some of the power and control differential.<br>• Using participative methodologies with young people can create the space for alternative understandings and "new discursive regimes" to emerge.<br>• Including young people in knowledge production disrupts traditional power differentials.<br>• Including young people as co-researchers facilitated the research.<br>• Young people's involvement with designing the methods may have helped facilitate far richer, engaging, and frank interviews.<br>• The methods included very flexible approaches to participation, allowing young people to participate in different ways (e.g., engage in different methods, participate in specific stages, withdraw from stages or whole project).	• The adult-child binary and its accompanying discourses and assumptions were constraints to working collaboratively with young people.
Goodley; 2022; UK (14)	Qualitative, text and opinion<br><br>Explore understanding of a construct; how disabled young people understand capital in their lives<br><br>Aged 14-36 years; male and female, including trans individuals; sought to	• Using images helped young people engage in the research.<br>• They shared models with young people early in the research process to promote co-production.	• COVID-19 impacted ways of working, including moving to working online. While potentially liberating, working online ignores disabled people's digital exclusion.

	include young people with learning disability and those identifying as men		
Hodson; 2022; Canada (15)	Text and opinion<br><br>Explore how participatory design can be more inclusive; supporting decision-making<br><br>Nursing students with learning disabilities (age not reported)	• Measures were put in place to promote the wellbeing of participants, particularly when their needs exceeded what the team was qualified to present. There was at least one local counsellor present for anyone who might need help.<br>• Power sharing was accomplished by having research teams that reflected the demographic of the user group and giving more agency to students and participants.<br>• Events were organized in locations familiar to participants for their emotional and physical accessibility and safety.<br>• The choice of methods used (personas) helped young people feel more at ease.	• Achieving genuine inclusion can be difficult.<br>• Involving users with complex needs or stakeholders working within complex systems can be difficult.<br>• Some groups of people may be overlooked in participatory design.<br>• In participatory design with "vulnerable groups" there is a reluctance to generalize best practices because of the diverse requirements of different groups.
Kendal; 2017; UK (16)	Qualitative<br><br>Develop participatory research method; emotional health support needs<br><br>Aged 16-18 years; predominantly female; most in full-time education	• The young people's analysis of their own data and contribution to the writing up process privileged their perspectives.<br>• The young person employed on the research team influenced all stages of the study.	• Challenges with recruitment left the authors with a smaller sample size than anticipated.
Knowles; 2022; UK, New Zealand (17)	Qualitative<br><br>Determine priorities for systematic review; co-design to design	• Using established networks to support young people to become involved in the review.<br>• Consider the requirements of different methods, and decide if researchers	• Participatory codesign is time-consuming.<br>• Time and collaboration are needed to determine how reviews should be influenced by co-designed processes.

	interventions<br><br>Aged 16-25 years; male, female, and non-binary young people; Māori young people included in New Zealand study; lived experience of both or either individual experience of self-harm or indirect experience of self-harm or suicide as a friend or family member	have the resources to implement them properly.<br>• While interactive debate and dialogue can be challenging, they provide a deeper exploration of patient and researcher perspectives.<br>• Using certain methods (e.g., Persona method) helped make it clear which parts of the review the researchers wanted to discuss.	• A large degree of reflexivity on behalf of the researchers may be necessary to respond to the challenges to ways of thinking raised by young people. This may be particularly challenging for researchers with less experience in qualitative or participatory research.<br>• The research process was time consuming.<br>• Implementing suggestions from participatory work may not be straightforward.
Lapadat; 2020; Canada (18)	Qualitative<br><br>Describe opportunities and challenges of co-collaboration; self-management for youth with bipolar disorder<br><br>Aged 20-25 years; male and female; had diagnosis of bipolar disorder	• Young people and researchers had different goals. Establishing spheres of leadership was a reflexive process that allowed researchers and young people to consider their goals in tandem: distinct, yet moving in the same direction.<br>• Relationships between community organizations and young people may be challenging (i.e., young people may be reliant on services provided by research or community organizations). The authors recommend continually reassuring participants that their relationship to services is not contingent on their research participation, and that the team's highest priority is that community members are not facing barriers to services.<br>• Continuous communication with young participants ensured they felt empowered and credited when desired while respecting their desire for confidentiality and anonymity.	• It was predicted that active mood episodes may affect the retention and participation of some Youth Action Group members. Some did leave the project for this reason.<br>• It is challenging to accommodate the fluctuating school and work schedules of youth.<br>• Youth may have felt unsure about what they were meant to do and therefore may not have prioritized the project. Establishing more concrete responsibilities early in the project may have helped youth become more engaged at earlier stages.<br>• Peer researchers may feel discouraged from contributing to discussions if they feel their input isn't valued. Creating an environment in which their thoughts and opinions are respected helps them to feel their input is legitimate.

		• Health professionals should be available should distress occur. Sensitive information should be carefully conveyed.<br>• Young people should be compensated for their time and effort, including through payment, reimbursement for expenses, providing meals and snacks, demonstrating respect and appreciation, and publicly acknowledging contributions. Monetary compensation reinforced member expertise and demonstrated the value of their contributions.<br>• Sharing meals helped to build trust and mutuality between young people and researchers.<br>• Establishing principles of work is foundational to creating positive working relationships. The process that emerged was continuous communication between meetings, frequent check-ins, specified deliverables, and clear deadlines.<br>• Longer timelines can provide space for capacity and confidence building among young people.<br>• Young people need space to ask questions and research their ideas to help them build capacity to collaborate as equals.<br>• Consistent contact (e.g., meeting reminders, communication about assigned work) can improve output and engagement.<br>• Assign concrete responsibilities and balance flexibility with expectations to help young people stay engaged.<br>• Reassure young people their input is valued and facilitate their input.	

		• Community partners can serve as a liaison between academics and young people, and aid them in expressing their views.<br>• Make sure young people understand how activities relate to the project aims. This can be accomplished by creating an open atmosphere where questioning was encouraged, conveying information through different means, opening discussions, and maintaining consistent communication.	
Lee; 2023; Australia (19)	Mixed methods<br><br>Describe a pilot evaluation; Lived Experience Working Group protocol<br><br>Aged 16-30 years; lived experience of mental ill-health		• Lack of time and funding hinder youth participation.
Liddiard; 2019a; UK (20)<br><br>"working the edges"	Qualitative<br><br>Co-produce theoretical knowledge; lives and aspirations of young people with life-limiting and life-threatening impairments<br><br>Aged 19-30 years; female; living with life-limiting and life-threatening impairments	• Using multiple virtual environments and social media platforms facilitated inclusion and accessibility.<br>• The authors drew on affect theory and the psychoanalytic notion of desire to consider relationships and break down power relations.	• Many people with disability are still digitally excluded; other forms of marginalization intersect with disability to create digital exclusion.

Liddiard; 2019b; UK(21)<br><br>“i was excited”	Qualitative<br><br>Describe co-produced disability research; living with life-limiting and life-threatening impairments<br><br>Aged 19-30 years; female; living with life-limiting and life-threatening impairments	• Online spaces could help build meaningful relationships with disabled young people.<br>• Participation and leadership were adapted to fit the needs and wants of the co-researchers.<br>• Co-researchers were heavily involved in decision-making and undertook most of the fieldwork.<br>• Reflection among the researchers regarding the approach and relationships necessary to recruit young people with disability to the project<br>• More practical and explicit information needs to be written into research studies to acknowledge the contributions of young people with disabilities, instead of simply negatively emphasizing the additional time and resources required to engage this group.<br>• The networks of co-researchers can help researchers access communities that may otherwise have been inaccessible.<br>• Establishing a budget to fund co-researchers to purchase technology as recognition of their commitment and work on the project.<br>• Invited co-researchers to become members of the research center, offer access to research communities and university certificates and references in recognition of their contributions and expertise.	• Recruiting co-researchers could be difficult.<br>• Situating sessions within school likely meant the project was perceived as another form of schoolwork.<br>• Young people were juggling multiple other responsibilities in addition to the project.<br>• There was a lack of diversity among young people involved in the project. Co-production may be an exclusive space even within disability research.

Liebenberg; 2017; Canada (22)	Qualitative<br><br>Exploring facilitating youth engagement; mental health<br><br>Aged 12-18 years; First Nations	• Working collaboratively with youth and community partner staff could amplify the dissemination of findings.<br>• The involvement of community partners who were members of communities participating in the research helped establish trusting relationships between young people, their families, and researchers.<br>• The more youth gained a sense of ownership of the research process, the more fully they could participate.<br>• Identifying safe spaces (e.g., a service provider's youth centre) gave young people and the research team a place to spend time around research activities.<br>• Choosing methods appropriate to the young people was important, i.e., using images to facilitate narratives on lived experience and cultural traditions of storytelling.<br>• The project used a continuous consent process. In connection with flexibility, this created a space where young people could become comfortable with the research process.<br>• Young people could assume as much or little ownership of the research process as they wanted (e.g., adapting methods).<br>• A research plan is needed to gain community permission to work with youth and community.	
Mannell; 2023; South Africa (23)	Qualitative<br><br>Challenging power relations in co-	• Creating spaces for constant reflection by the research team allowed for challenging research epistemologies.<br>• Embracing differences and conflicts	• Open disagreement between youth researchers and research team was challenging for the research team. The two groups had different

	production; HIV and intimate partner violence prevention<br><br>Aged 18-29 years; men and women; living in informal settlements and rural communities; not currently in school or formal work	that arise between researchers' perspectives and those of people with lived experience may improve coproduction.<br>• Research capacity was strengthened by involving end users in the interpretation of data and by explaining research concepts in a way that was meaningful to them. Interpretive ownership of data was considered a key principle for successful coproduction.<br>• Trust was built through small group work with similar individuals and trust-building activities facilitated by experts on the research team.<br>• The authors selected their methods to facilitate more open conversations with young people about their lived experience and to strengthen young people's capacity in understanding research.	interpretations of the data and therefore different interpretations of what was most important to address as part of the intervention.<br>• Young people were hesitant to discuss aspects of their lives that they thought the research team might not understand, like drug use or violence in their relationships.<br>• Young people were cautious of sharing personal information with one another; it took months for them to openly discuss their personal lives in a group.<br>• Young people, as paid employees, were concerned about how illegal and negative behaviours would impact on their involvement in the project.
Mawn; 2015; UK (24)	Qualitative<br><br>Describe establishment of youth research group; mental health<br><br>Aged 14-24 years		• Identifying young people who want to be involved in research can be a time consuming and difficult process.<br>• There are significant costs to delivering projects that develop a youth culture given the need to utilize youth friendly locations, travel costs for members, youth friendly refreshments, activities, training, and mentoring. When considering young people and sustainable engagement over time, fun activities such as team building days are important but rarely the focus of research grant applications.<br>• Poor involvement of young people can lead to a loss of self-confidence and a negative view of research.

			• Supporting young people to be involved in research can be expensive and difficult to organize.<br>• The authors anticipated young people will come and go from the group (primarily due to lifestyle changes and developmental needs). Minimizing disruption caused by this is key to the success of the group, thus on-going recruitment is necessary.<br>• Young people have complex and dynamic lives. Young people often balance education, part-time employment, sport, and social activities.<br>• Lack of funding is the primary concern and threat to the sustainability of their project, and this is compounded by a lack of specific funding initiatives that are focused on public and patient involvement, which will fund ongoing, sustained involvement beyond just a discrete research project.<br>• Research involving young people can be difficult to navigate through research ethics committees.
McCabe; 2023 (25)	Systematic review<br><br>Describe impacts of young engagement; mental health research<br><br>Aged 8-25 years for included studies in review<br><br>Youths aged 19-24	• Building relationships with community organizations [to do what e.g. recruitment]?<br>• Reflexivity in adult researchers with respect to an awareness of power dynamics, how they are relating with youth<br>• Clear expectations for youth about engagement<br>• Having diversity among youth voices<br>• Use of pre- and debriefs for large	• Monitoring whether youth are remaining representative of young people. As youth researchers become more involved in the project they may begin to think more like adult researchers.<br>• Sustaining engagement over the course of the project.<br>• Balancing bringing together a diversity of backgrounds and perspectives versus efficiency in

	years acted as consultants for review; male; Canadian citizens	meetings<br>• Flexibility with degree of involvement and scheduling<br>• Build relationships with refreshments and ice-breaking activities<br>• Have a dedicated youth engagement coordinator<br>• Using youth friendly communication tools<br>• Using accessible language<br>• Power sharing with youth i.e., empower in decision making, treating youth as equals.<br>• Overcoming power differentials between youth and adult researchers was identified by the youth as key to fostering meaningful collaboration.<br>• Efforts to build relationships (genuine, trusting) between youth and adult researchers.<br>• Young people involved in the review felt that adult researchers engaging with youth in a mental health context should have training in trauma-informed approaches as well as cultural competence. Asking youth to share their experiences may be retraumatizing or cause them significant distress.	decision-making.<br>• Recruitment of youth researchers (e.g., finding appropriate youth, representing diversity)<br>• Managing youth expectations (e.g., about the impact of the project)<br>• More time/effort to build relationships, especially in mental health which can be a sensitive issue<br>• Power imbalances between youth and adults<br>• Not involving youth early enough to influence the project<br>• More work to support (e.g., training, accommodating needs) and coordinate youth engagement<br>• More work to set up engagement (as a new process)<br>• Navigating diverse perspectives and conflicting priorities (adult vs youth, youth vs parents)<br>• Communication barriers between adult and youth researchers<br>• Navigating research ethics boards<br>• Lack of time: more funding, time, work
Mulvale; 2019; Canada (26)	Qualitative<br><br>Evaluation of elicitation techniques; mental health care, transitions to adult care, employment supports<br><br>Aged 16-25 years;	• Relationships and communication between participants and researchers are fundamental to success<br>• Skillful facilitation was needed to balance the time needed for full discussions, so people felt valued with the need to develop concrete solutions.<br>• Time was needed undertake research activities (e.g., create videos, co-design solutions) and to share	• Sustaining meaningful youth engagement was challenging, as young people faced competing demands for their time and attention in addition to struggles with ongoing mental health issues.<br>• Overcoming power imbalances may be challenging, and young people may not want to share their perspectives in an environment with family members

	male and female; experiencing mental health problems	perspectives, build trust, and create group cohesion.<br>• Created a warm, welcoming environment where everyone was heard and had a voice, and no ideas were quashed.<br>• Created a collective vision and an environment where young people and research team were "all in this together." Valued hearing from others, particularly youth.<br>• Shared power by following techniques of deliberative dialogue, recognizing that "conversation works best instead of being talked to or talked at."<br>• Building trust as an important first step of the process. Trust needed to be enhanced and sustained.<br>• Youth need to feel that their input is not tokenistic and that they are really being listened to, or they may disengage.<br>• Using a number of different elicitation techniques helped young people "find their voice."	and service providers.
Norton; 2021(27)	Systematic review<br><br>Review evidence on concept of co-production; co-production in child and adolescent mental health<br><br>Children and adolescents (age not reported)	• Creating an environment that allows individuals to discuss and work through past traumas without dismissing them is vital to co-productive work<br>• It is important to consider an environment that is conducive to recovery by: considering participants as assets rather than passive recipients of services; developing capacity by moving from deficit to strength-based; encouraging mutuality in traditional relationships; blurring traditional boundaries that separate service providers from service users; creating	

		network development to enable the transfer of knowledge between partners; facilitating rather than deliver services	
Pavarini; 2019; UK (28)	Text and opinion<br><br>Provide model of young people's advisory group; guidance on co-production process<br><br>Aged 15-18 years; shared interest in ethics and mental health; included individuals with mental health difficulties and from socially marginalized groups or with special needs	• Different interests and voices should be represented in the groups of young people. This should be considered during the recruitment process and running of group sessions.<br>• Developing a set of collective principles of work for facilitators and youth (e.g., responsibility, responsivity, transparency, empathy, confidentiality) creates clear structure.<br>• Collectively drafting a 'contract' for participants and facilitators to manage expectations and priorities and to understand shared values and principles in the initial meeting.<br>• Keeping similar schedules for each meeting and having appropriate, well-equipped facilitators<br>• Offering optional activities for youth on top of regular meetings<br>• Selecting young people "that will most benefit the group – and from the group"<br>• Researchers reflecting on an ongoing basis on the effects of the group on young people and research<br>• Providing different roles and additional support for different participants depending on strengths and interests<br>• Need to continually document changes in the project<br>• Researchers should know how and	• Ensuring the protection of children and young people, particularly those with lived experience of mental health challenges, required the development of tailored approaches, which added complexity to the co-design process.

		why they are using co-production, and should have a substantial and transparent justification for involving young people in the research process.<br>• Anonymous assessment questionnaires can help researchers understand young people's priorities and what "works" and what doesn't in the coproduction process. Researchers can then make changes in response to feedback e.g., changing structure of sessions.	
Ramey; 2019; Canada (29)	Qualitative<br><br>Evaluation of advisory group; participatory research evaluation<br><br>Aged 15-29 years	• Shared power by following techniques of deliberative dialogue, recognizing that "conversation works best instead of being talked to or talked at."<br>• Building trust as an important first step of the process. Trust needed to be enhanced and sustained.<br>• Maintain continuity of youth involvement.<br>• Take the time to build relationships and have face to face meetings.<br>• Value different forms of knowledge and experience, including the expertise that is lived experience<br>• Communicate appropriately so that knowledge is translated in a way that is easily understood and applied<br>• Embrace the unexpected, including mutual respect for everyone's time and having generous timelines and being patient with each other and with ambiguity<br>• Provide clear structure and support<br>• Continually revisit and renegotiate structure and flexibility	• Challenges defining shared responsibilities between youth and adults<br>• Structure and clarity of roles seen as a challenge due to the ambiguous nature of a Living Laboratory<br>• Difficult coordinating a national roundtable across a large country
Robinson; 2023; Australia (30)	Qualitative	• Concentrated mentoring and supervision facilitated the co-	

	Exploring challenges to centering young people's voices; lived experience of family violence<br><br>Included 1 young co-researcher with lived experience of significant disability	researcher to do her work<br>• Ensuring accessible modes of work (e.g., reduced and flexible working hours) facilitated the co-researcher to do her work	
Rome; 2015; UK (31)	Qualitative<br><br>Explore transitions in schooling and services; transition services for young people with disability and adult-free research spaces for young people<br><br>Aged 12-19 years; lived experience of disability; male and female	• Running events in an informal, accessible venue that is familiar to young people is important to put people at ease.<br>• Creating research spaces, including adult-free spaces, for young people to initiate and conduct their own research in ways that are manageable and meaningful to them.	• The authors state adult attitudes were the biggest challenge by far. Adults often have very limited ambitions and aspirations on behalf of this group of young people and did not think they were capable of contributing to the discussions about Transitions services, never mind carrying out their own piece of research or making their own DVD.<br>• Budget/finances<br>• Transport in the area was generally very difficult, and public transport for most of their group was not possible. Getting the young researchers to meetings was a "logistical headache."<br>• The cost of taxis, support workers, and volunteers for the young researchers
Sellars; 2021; UK (32)	Scoping review<br><br>Review youth-focused health studies; use of young people's advisory groups in health research<br><br>Aged 12-18 years;	• Having a dedicated staff person who is responsible for supporting the young people and answering questions from researchers about youth participation	• Challenges in accessing young people<br>• Youth participation not seen as part of workplace culture<br>• Lack of resources for genuine youth participation<br>• Lack of understanding of how youth can participate and be engaged in research<br>• Delays if ethical approvals or

	lived experience of mental health challenges; all were members of NeurOX YPAG		safeguards are needed
Soleimanpour; 2008; United States (33)	Qualitative<br><br>Reflections on youth-led research; school health programs<br><br>Middle and high school students (grades 8-11) (age not reported); female and male; represented racial and ethnic diversity of school	• Youth felt validated and valued when provided with meaningful venues to share their research.<br>• Researchers need to be realistic about the research that can be conducted.<br>• Define the decision-making power of each partner in the research at the beginning, and define it clearly at each point of the research process.<br>• Engaging student research team members in staff meetings and allowing them to report regularly to the staff and get feedback helped to guarantee that the student's efforts were integrated into the project<br>• To keep youth engaged, adult allies supported the youth in creating a clear vision and timeline for the full scope of their projects.<br>• To ensure that the research process was meaningful, it was essential that the student research teams partnered with all relevant parties from the onset.	• The length of projects was not enough to enable projects to finish.<br>• Challenging working with a diverse group of young people, reconciling different attitudes and opinions, and for young people to learn to speak up.<br>• Students reported challenges mastering new research skills.<br>• Poor attendance as young people juggled numerous responsibilities.
Spears; 2021; Australia (34)	Qualitative<br><br>Describe participatory design processes; health campaigns and bullying prevention<br><br>Young people (age not reported)		• Researchers and adults need to be open to relinquishing power to young people<br>• Researchers need to be flexible, particularly when considering hybrid environments.<br>• Engaging in participatory design can be unpredictable and time consuming.

Spuerck; 2023; UK, India, Pakistan, Turkey, Kenya, South Africa, Brazil, Portugal (35)	Qualitative<br><br>Explore how to meaningfully involve young people in research; barriers and enables of peer researcher roles in mental health<br><br>Aged 14-24 years; lived experience of depression and anxiety	• Young participants felt more comfortable working with a peer, rather than a career researcher. Co-facilitating focus groups between a peer and career researcher helped to achieve this balance.	• The recruitment of peer researchers with lived experience of mental health difficulties can be more challenging than in non-mental health related research. Young people might fear their involvement could face stigmatizing attitudes from the research team or stakeholders, and lead to the deterioration of existing or the recurrence of previous mental health difficulties<br>• Engaging with young people could be challenging as there were sociocultural barriers even within the same society.<br>• Facilitating focus groups could be difficult, as young people needed to be kept engaged while encouraging them to open up on sensitive experiences. This also involved handling different opinions and moderating between more vocal and quiet participants.<br>• The terms peer advisor and peer researcher are not easy to understand and translate in other languages so they should be avoided.<br>• Allocated time was insufficient.
Swist; 2022; Australia (36)	Qualitative<br><br>Document research process; establish adolescent health research community of practice<br><br>Aged 13-26 years; males and females from diverse cultural	• Sustaining trusting relationships requires transparency and flexibility based on a co-designed partnership model so that clear expectations can scaffold present and future collaborations.<br>• Longer-term commitments with young people are vital to support health research capacity-building and priority-setting beyond short-term funding cycles.	• Lack of understanding of the views of adult stakeholders regarding youth engagement

	backgrounds		
Taggart; 2022; UK (37)	Qualitative<br><br>Develop training program; service assessment and training implementation<br><br>Project aimed at those aged 16-25 years; had experience of complex mental health needs	• The experience of working with young people who were experiencing mental health problems meant that the professionals in the project team needed to be mindful of their (young people's] mental health needs.<br>• Welcoming critical perspectives<br>• Spelling out the nature of participation in all project documents (e.g., spelling out that young people could have complete editorial and creative control about training content while professionals could make choices about delivery).<br>• Important to have a clear supervision structure and think through issues related to boundaries (e.g., different working relationships forged between young people and staff).<br>• Being inclusive of diverse mental states and mental health crises (e.g., not just including people who were recovered in the work)<br>• Having unstructured and unpressured discussion sessions that could be fun and creative<br>• Flexibility with respect to time, engaging with young people on their terms and with their needs in mind, holding meetings in a more neutral environment (i.e., local community college)	• Engaging with young people experiencing mental health crises and challenges can be challenging (e.g., navigating pick up and drop off from inpatient services).<br>• Ethical dilemma related to the paradox of young people's experiences being seen as evidence of mental illness in one context and legitimate experience in another context<br>• The authors' project did not work to organizational deadlines and the project overran<br>• Some professionals who were working on the project also worked in teams that provided services to some of the young people involved in the project.<br>• Support needs should be considered fully. Young people also required support to attend meetings beyond what was initially expected.
Taylor; 2022; UK (38)	Qualitative<br><br>Define research priorities; youth mental health	• Collaborating across academic disciplines and stakeholder groups<br>• Building on people's existing capabilities<br>• Used conversation templates to facilitate dialogue between the	• Stakeholders may have different priorities and need to have difficult conversations to discuss the limitations and possibilities for the research.<br>• Young participants may lack

	Aged 16-24 years; with care experiences and/or identifying as LGBTQ+	different stakeholders in order to hear each other's thoughts, explore similarities and differences, and arrive at a set of priorities that reflect the full range of experiences and viewpoints.<br>• Seeing people for 'who they are' i.e., recognizing their intersectional experiences<br>• Investing significant staff time in building relationships between staff and YAG members<br>• Having clear processes for building young people's capacity to participate (as part of the YAG)<br>• Ensuring young people and other stakeholders were involved early as partners in setting the research agenda	confidence to speak up in workshops.
Thomson; 2022; UK (39)	Qualitative<br><br>Explore engagement in research process; Patient and Public Involvement program development<br><br>Aged 16-24 years, lived experience of mental illness	• Limiting number of participants in sessions to ensure discussion could be coordinated and using relatable facilitators of a similar age to participants<br>• Allowing young people to have input on research priorities<br>• Adapting sessions to meet needs of group members (e.g., ice breakers, reflecting on discussion at last meeting)<br>• Developing a 'Researcher Agreement' to ensure mutual respect.<br>• Important to be aware of the emotional experience connected to research. Offering knowledge based on lived experience can potentially be triggering.<br>• When working with young people, this should be a collaborative and respectful environment with achievable, non-tokenistic goals.	• Researchers need to reflect on their own practice and ensure they are working to create an environment where young people can make meaningful contributions to mental health research.<br>• The materials used within the group may need to differ according to age. Need to ensure that the language used in resources is accessible to all young people.<br>• Lack of funding hindered the expansion of paid positions on the research team and precluded expanding the scope of the project and hiring youth as staff.<br>• Lack of digital skills or lack of access to technology was a barrier to working virtually during COVID.<br>• No ethics approval was required for patient public involvement, so it can be implemented in a tokenistic

			manner.
Woods-Jaeger; 2022; United States (40)	Qualitative<br><br>Develop prevention strategy; health inequities and racism<br><br>Ages 12-17 years; men and women; Black youth	• Engaging with key community partners, who served as a bridge to build trust, credibility, and integrity in the process. They aided in recruitment and session facilitation efforts, enabling the critical engagement of youth in a virtual environment. Due to their knowledge of community resources and opportunities, our community partners were also vital in leveraging resources and opportunities to build youth agency, leadership capacity, advocacy skills, and political efficacy for social action.<br>• It is recommended that prevention scientists form diverse teams and invest the time and resources to form equitable youth partnerships that sustain over time. To translate science into sustainable change, it is critical that prevention scientists continue to build collective power (e.g., networks, resources and structures) that last beyond a specific grant. It is recommended that prevention scientists and community partners proactively plan for sustained engagement in social action planning that extends beyond current funding and the tenure of current research and youth partners.<br>• To facilitate trust building between youth and academic researchers, the researchers promoted youth ownership of the research and action process. To promote youth ownership, it is critical that prevention scientists recognize, give space, and honor youth as experts	• Lack of funding continuity<br>• In initial sessions, youth reported a lack of familiarity with one another as well as the academic partners.

		in their own lived experiences.<br>• A variety of methods were used by young people in the project to help them engage in reflection and identity possible mechanisms for action.	
Wright; 2019; Australia (41)	Qualitative<br><br>Explore engagement with services; youth mental health services<br><br>Aged 15-34 years; Aboriginal and Torres Strait Islander young people living on Wadjuk Nyoongar country (Perth, WA)	• Investing in establishing safe spaces for the Elders, young people, and service providers to engage in open, honest dialogue.<br>• Safe spaces in this context includes service providers having a heightened awareness of their role and actively addressing power imbalance between young people and themselves.<br>• Safe spaces is about ensuring the voices of the young people and the Elders are heard and respected.<br>• Applying a decolonizing framework. The first engagement activity was an On Country event, placing Nyoongar culture at the center of the engagement and disrupts service providers' typical ways of working.	
Zlotowitz; 2016; UK (42)	Qualitative<br><br>Ethnography of co-produced intervention; building relationships to address needs<br><br>Aged 16-22 years; predominantly male; living in inner city housing estate; primarily white British ethnicity; identified by community agencies as gang-affiliated	• Young people sought support from practitioners during sessions. Young people wanted resources, opportunities, and contacts to help them move from risky contexts to safer ones.<br>• It was important for the project to be peer and youth led wherever possible.<br>• Young people were clear that word of mouth was the strongest referral system and that they would bring friends along if practitioners were deemed useful.<br>• Young people needed youth-led activities to be very local, within their	• Frustration among young people about local services and community resources created challenges for practitioners in engaging them with these agencies, despite efforts to support young people in envisioning and advocating for systemic change.<br>.

		‘safe territory’, and for practitioners to come to them.<br>• Young people wanted responsive, flexible, and relevant activities that those chose to engage in.<br>• Young people were clear that long-term and consistent relationships with practitioners who seem to genuinely care, were non-judgemental, and whom they trusted were core to engagement. Young people were then able to share their emotional experiences.	

1. Ali AZ, Wright B, Curran JA, Newton AS. Review: Patient engagement in child, adolescent, and youth mental health care research – a scoping review. Child Adolesc Ment Health. 2023;28(4):524–35.

2. Bennett V, Gill C, Miller P, Wood A, Bennett C, Ypag NO, et al. Co-production to understand online help-seeking for young people experiencing emotional abuse and neglect: Building capabilities, adapting research methodology and evaluating involvement and impact. Heal Expect. 2022;25(6):3143–63.

3. Bennett V, Gill C, Miller P, Lewis P, Ypag NO, Hamilton-Giachritsis C, et al. Developing a novel co-produced methodology to understand 'real-world' help-seeking in online peer–peer communities by young people experiencing emotional abuse and neglect. Heal Expect. 2022;25(6):3124–42.

4. Boswell N, Woods K. Facilitators and barriers of co-production of services with children and young people within education, health and care services. Educ Child Psychol. 2021;38(2):41–52.

5. Brady G, Franklin A. Challenging dominant notions of participation and protection through a co-led disabled young researcher study. J Child Serv. 2019;14(3):174–85.

6. Canas E, Lachance L, Phipps D, Birchwood CC. What makes for effective, sustainable youth engagement in knowledge mobilization? A perspective for health services. Heal Expect. 2019;22(5):874–82.

7. Culbong T, Crisp N, Biedermann B, Lin A, Pearson G, Eades AM, et al. Building a Nyoongar work practice model for Aboriginal youth mental health: prioritising trust, culture and spirit, and new ways of working. Heal Sociol Rev. 2022;31(2):173–92.

8. Davison J, Maguire S, McLaughlin M, Simms V. Involving adolescents with intellectual disability in the adaptation of self-reported subjective well-being measures: participatory research and methodological considerations. J Intellect Disabil Res. 2022;66(7):628–41.

9. Dewa LH, Lawrence-Jones A, Crandell C, Jaques J, Pickles K, Lavelle M, et al. Reflections, impact and recommendations of a co-produced qualitative study with young people who have experience of mental health difficulties. Heal Expect. 2021;24(S1):134–46.

10. Dewa LH, Crandell C, Choong E, Jaques J, Bottle A, Kilkenny C, et al. CCopeY: A Mixed-Methods Coproduced Study on the Mental Health Status and Coping Strategies of Young People During COVID-19 UK Lockdown. J Adolesc Heal [Internet]. 2021;68(4):666–75. Available from: https://doi.org/10.1016/j.jadohealth.2021.01.009

11. Dunn V. Young people, mental health practitioners and researchers co-produce a Transition Preparation Programme to improve outcomes and experience for young people leaving Child and Adolescent Mental Health Services (CAMHS). BMC Health Serv Res. 2017;17(1):1–

12.

12. Edwards P, Bunn H. ‘We have to carry on’. A participatory research project with young people enrolled on a supported internship programme during the Covid-19 pandemic. Educ Child Psychol. 2022;39(3):56–69.

13. Fox R, Nic Giolla Easpaig B, Watson L. Making Space for Community Critical Methodology: Stories from the Australian Context. Am J Community Psychol. 2019;63(1–2):227–38.

14. Goodley D, Liddiard K, Runswick-Cole K, Watts L, Whitney S, Dobbin H, et al. Posthumanist disability studies. Palgrave Handb Crit Posthumanism. 2022;2:793–822.

15. Hodson E, Svanda A, Dadashi N. Whom do we include and when? participatory design with vulnerable groups. CoDesign [Internet]. 2023;19(4):269–86. Available from: https://doi.org/10.1080/15710882.2022.2160464

16. Kendal SE, Milnes L, Welsby H, Pryjmachuk S, Shafeeah C, Annaruth D, et al. Prioritizing young people’s emotional health support needs via participatory research. J Psychiatr Ment Health Nurs. 2017;24(5):263–71.

17. Knowles S, Sharma V, Fortune S, Wadman R, Churchill R, Hetrick S. Adapting a codesign process with young people to prioritize outcomes for a systematic review of interventions to prevent self-harm and suicide. Heal Expect. 2022;25(4):1393–404.

18. Lapadat L, Balram A, Cheek J, Canas E, Paquette A, Michalak EE, et al. Engaging youth in the Bipolar Youth Action Project: Community-based participatory research. J Particip Med. 2020;12(3).

19. Lee GY, McKenna S, Song YJC, Hutcheon A, Hockey SJ, Laidler R, et al. Strengthening mental health research outcomes through genuine partnerships with young people with lived or living experience: A pilot evaluation study. Heal Expect. 2023;26(4):1703–15.

20. Liddiard K, Whitney S, Evans K, Watts L, Vogelmann E, Spurr R, et al. Working the edges of Posthuman disability studies: theorising with disabled young people with life-limiting impairments. Sociol Heal Illn. 2019;41(8):1473–87.

21. Liddiard K, Runswick-Cole K, Goodley D, Whitney S, Vogelmann E, Watts MBE L. “I was Excited by the Idea of a Project that Focuses on those Unasked Questions” Co-Producing Disability Research with Disabled Young People. Child Soc. 2019;33(2):154–67.

22. Liebenberg L, Sylliboy A, Davis-Ward D, Vincent A. Meaningful Engagement of Indigenous Youth in PAR: The Role of Community Partnerships. Int J Qual Methods. 2017;16(1):1–11.

23. Mannell J, Washington L, Khaula S, Khoza Z, Mkhwanazi S, Burgess RA, et al. Challenges and opportunities in coproduction: Reflections on working with young people to develop an intervention to prevent violence in informal settlements in South Africa. BMJ Glob Heal. 2023;8(3):1–9.

24. Mawn L, Welsh P, Stain HJ, Windebank P. Youth Speak: Increasing engagement of young people in mental health research. J Ment Heal. 2015;24(5):271–5.

25. McCabe E, Amarbayan M, Rabi S, Mendoza J, Naqvi SF, Thapa Bajgain K, et al. Youth engagement in mental health research: A systematic review. Heal Expect. 2023;26(1):30–50.

26. Mulvale G, Moll S, Miatello A, Murray-Leung L, Rogerson K, Sassi RB. Co-designing Services for Youth With Mental Health Issues: Novel Elicitation Approaches. Int J Qual Methods. 2019;18:1–13.

27. Norton MJ. Co-production within child and adolescent mental health: A systematic review. Int J Environ Res Public Health. 2021;18(22).

28. Pavarini G, Lorimer J, Manzini A, Goundrey-Smith E, Singh I. Co-producing research with youth: The NeurOx young people's advisory group model. Heal Expect. 2019;22(4):743–51.

29. Ramey HL, Rayner ME, Mahdy SS, Lawford HL, Lanctot J, Campbell M, et al. The Young Canadians Roundtable on Health: promising practices for youth and adults working in partnership. Can J Public Heal. 2019;110(5):626–32.

30. Robinson S, Foley K, Moore T, Valentine K, Burton J, Marshall A, et al. Prioritising Children and Young People with Disability in Research About Domestic and Family Violence: Methodological, Ethical and Pragmatic Reflections. J Fam Violence. 2023;38(6):1191–204.

31. Rome A, Hardy J, Richardson J, Shenton F. Exploring transitions with disabled young people: Our experiences, our rights and our views. Child Care Pract. 2015;21(3):287–94.

32. Sellars E, Pavarini G, Michelson D, Creswell C, Fazel M. Young people's advisory groups in health research: Scoping review and mapping of practices. Arch Dis Child. 2021;106(7):698–704.

33. Soleimanpour S, Brindis C, Geierstanger S, Kandawalla S, Kurlaender T. Incorporating youth-led community participatory research into school health center programs and policies. Public Health Rep. 2008;123(6):709–16.

34. Spears BA, Taddeo C, Ey LA. Using participatory design to inform cyber/bullying prevention and intervention practices: Evidence-

Informed insights and strategies. J Psychol Couns Sch. 2021;31(2):159–71.

35. Spuerck I, Stankovic M, Fatima SZ, Yilmas E, Morgan N, Jacob J, et al. International youth mental health case study of peer researchers' experiences. Res Involv Engagem [Internet]. 2023;9(1):1–11. Available from: https://doi.org/10.1186/s40900-023-00443-4

36. Swist T, Collin P, Nguyen B, Davies C, Cullen P, Medlow S, et al. Guiding, sustaining and growing the public involvement of young people in an adolescent health research community of practice. Heal Expect. 2022;25(6):3085–95.

37. Taggart D, Nye C, Taylor J, Solstice J, Harrison M, Bryant R, et al. The Define Normal Project. Palgrave Handb Innov Community Clin Psychol. 2022;239–57.

38. Taylor A, McMellon C, French T, MacLachlan A, Evans R, Lewis R, et al. Defining research priorities for youth public mental health: reflections on a coproduction approach to transdisciplinary working. Heal Res Policy Syst [Internet]. 2022;20(1):1–14. Available from: https://doi.org/10.1186/s12961-022-00871-w

39. Thomson A, Peasgood E, Robertson S. The Youth Patient and Public Involvement Café—A youth-led model for meaningful involvement with children and young people. Heal Expect. 2022;25(6):2893–901.

40. Woods-Jaeger B, Jahangir T, Lucas D, Freeman M, Renfro TL, Knutzen KE, et al. Youth Empowered Advocating for Health (YEAH): Facilitating Partnerships Between Prevention Scientists and Black Youth to Promote Health Equity. Prev Sci [Internet]. 2024;25(1):20–30. Available from: https://doi.org/10.1007/s11121-022-01450-9

41. Wright M, Culbong T, Crisp N, Biedermann B, Lin A. "If you don't speak from the heart, the young mob aren't going to listen at all": An invitation for youth mental health services to engage in new ways of working. Early Interv Psychiatry. 2019;13(6):1506–12.

42. Zlotowitz S, Barker C, Moloney O, Howard C. Service users as the key to service change? The development of an innovative intervention for excluded young people. Child Adolesc Ment Health. 2016;21(2):102–8.
