# Supplementary material for: Collaborating With Young People: Identifying the Barriers and Facilitators in Co‐Designed Research
Source: Health Expect. 2025 May 27;28(3):e70308. doi: 10.1111/hex.70308 (PMC12117193; doi:10.1111/hex.70308)
Supplement: Supplementary file 3 — Appendix 1 Search strategy 20 8 2024. [file HEX-28-e70308-s002.pdf]

Appendix 1: Search strategy

The search strategy was amended as required for other databases searched.

Scopus

Date searched: 23 June 2023

#	**Searches**
1	("co-design" OR codesign OR "co-production" OR coproduction OR "participatory design" OR "participatory research" AND "young people" OR youth* OR adolescent* AND disabil* OR "practice based" OR "mental health") AND PUBYEAR > 2002 AND PUBYEAR < 2024
2	(LIMIT-TO (LANGUAGE , "English"))
3	(LIMIT-TO (DOCTYPE , "ar") OR LIMIT-TO (DOCTYPE , "re") OR LIMIT-TO (DOCTYPE , "ch"))
